# Analytical Performance of Electromembranes as a Tool for Nanoconcentrations of Silver in Waters

**DOI:** 10.3390/membranes13010011

**Published:** 2022-12-21

**Authors:** Macarena Silva, Carolina Mendiguchía, Carlos Moreno

**Affiliations:** Department of Analytical Chemistry, Faculty of Marine and Environmental Sciences, University of Cadiz, 11510 Puerto Real, Spain

**Keywords:** electromembranes, silver, tri-isobutylphosphine sulphide, hollow-fibre liquid-phase microextraction, Draper–Lin design, seawater

## Abstract

Electromembranes increase the efficiency of metal transport in liquid-phase microextraction systems by applying an electric potential, which accelerates the transport. Nevertheless, to get high extraction percentages in short extraction times it is necessary to take into account a great variety of factors, and multivariate optimization techniques are the best alternative to determine the most influential variables and to optimize the extraction process. In this work, a fractional factorial design was applied to determine the most influential variables in the extraction of silver by electromembranes. Thus, the effect of tri-isobutylphosphine sulphide (Cyanex 471x) concentration in the organic solution, sodium thiosulphate concentration in the acceptor solution, nitrate concentration in the sample solution, extraction time, stirring rate and electric potential on the enrichment factor were studied. Once the most important variables were selected, a small composite design (Draper–Lin) was used to obtain their optimal values to maximize the enrichment factor. Under these conditions, an experimental enrichment factor of 49.91 ± 3.95 was achieved after 22 min. Finally, the effect of saline matrix on the enrichment factor was tested and the optimized system was successfully applied to analyse silver concentrations at ultratrace levels, within the range of 7–29 ng·L^−1^ in different real seawater samples.

## 1. Introduction

In recent decades, electromembrane extraction (EME) has been proved as a highly efficient preconcentration/separation method for the analysis of a variety of substances in pharmaceutical, environmental and medical samples [1,2]. The most noticeable advantages in comparison with other microextraction systems are related to the very short extraction times without reduction of enrichment factors [3,4]. Although it is evident that the application of an electric potential to microextraction systems improves the efficiency of extraction, optimization of the most influential variables in the enrichment factor is necessary to reach the best results [5]. Undoubtedly, there are a lot of variables influencing the analyte extraction by EME, such as the type of solvent, the carrier, the pH of the donor and acceptor solution, the stirring rate, the extraction time, etc. [5,6]. Nevertheless, the importance of each one to the overall transfer of the analyte is not clear since the optimization of these systems is usually performed using a univariate scheme. To clarify the importance of each variable and their interactions, experimental design can be the best alternative due to the high number of variables involved.

Usually, the optimization based on experimental designs is performed by a two-step process. First, a screening design such as full or fractional factorial allows the identification of the most influential variables and, afterwards, other approaches such as central composite or Box–Behnken designs allow the establishment of the critical levels for those influential variables [7]. Although experimental designs based on factorial design and response surface methods have demonstrated their practicality in optimizing liquid–liquid microextraction methods, their application in microextraction methods is still scarce [8]. In the case of EME, application of the Box–Behnken design and central composite design are the most used approaches to get the optimum values for the influential variables [9,10,11,12,13,14].

It must be noted that electromembranes have been mostly applied to the analysis of organic compounds and only a handful of papers deal with the analysis of metals using this methodology [15,16,17,18,19,20,21,22,23,24,25]. The first works in this field were focused on biological samples [15,16] and, a few years later, the first paper on the analysis of metals in water samples appeared [26]. However, EME applications to real water samples are still very scarce and almost unavailable in the case of seawater, with only a couple of applications to metal analysis in real seawater samples [17,27].

In addition, EME has been not applied to the analysis of silver in natural waters although it could solve some drawbacks found when hollow-fibre liquid-phase microextraction (HF-LPME) systems are used to determine this element in water samples. For example, extraction time in HF-LPME systems is still very high (up to 240 min) for routine analysis [28,29], and quantification of silver concentration in real seawater samples requires preparation of standard solutions in a synthetic seawater matrix and their treatment using the same extraction procedure as for the samples, increasing the operation time and the complexity of the procedure [30,31].

In this work, an evaluation of the most influential variables on silver extraction using EME has been performed using a two-step process. First, these variables were identified using a fractional factorial design and, afterwards, optimal conditions were obtained from a Draper–Lin small response surface design. Then, the applicability of the system to extract silver from saline samples was evaluated and the analysis of ultra-trace silver concentrations in real seawater samples was successfully performed.

## 2. Experimental Section

### 2.1. Reagents and Solutions

Silver standard solutions were prepared from a 1000 mg L^−1^ commercial standard solution obtained from SCP Science (Clark Graham, Canada). Potassium nitrate and sodium thiosulphate were purchased from Merck (Darmstadt, Germany). All the synthetic solutions were prepared with deionized water provided by a Quantum Ultrapure water supplier from Millipore (Bedford, MA, USA). Nitric acid (65%) from Panreac (Barcelona, Spain) was used to clean laboratory supplies. Kerosene from Sigma Aldrich (St. Louis, MO, USA) and 1-octanol from Panreac were used to prepare organic solutions of tri-isobutylphosphine sulphide (Cyanex 471x ©) from Cytec Industries Inc. (Woodland Par, NJ, USA). Argon for atomic spectroscopy was supplied from Air Liquide (Madrid, Spain).

### 2.2. Equipment

A polypropylene porous hollow-fibre Accurel PP S6/2 from 3M-Membrana (Wuppertal, Germany) with an inner diameter of 1800 µm, pore size of 0.2 µm, 70% of porosity and a wall thickness of 450 µm was used as support for the organic solution. A soldering gun from Crison (Barcelona, Spain) was used to seal the fibre end and the acceptor phase was introduced into the lumen of the fibre by a 100 µL micro-syringe from Hamilton (Bonaduz, Switzerland). Platinum electrodes (0.25 mm thick) from Sigma-Aldrich were connected to a power supply from Delta Electrónica (Madrid, Spain) for the application of an electric potential (0–300 V). During the extraction process, the electric current was continuously monitored with a UNIT-UT 33D digital multimeter from Madrid Electrónica (Madrid, Spain). After extraction, an atomic absorption spectrometer Analyst 800 from Perkin Elmer (Waltham, MA, USA) with graphite furnace atomizer (GFAAS) was used to determine silver in the acceptor solution.

### 2.3. EME Procedure

The new EME system is based on a previous HF-LPME system described elsewhere [29]. In this system, the transport mechanism was based on the formation of a neutral ion pair [(AgCyanex471)^+^ NO_3_^−^] which can be solubilized into the organic solution. Cyanex 471x is a selective extractant for silver separation and, once into the organic–acceptor solution interface, the ion pair reacts with the acceptor agent (S_2_O_3_^2−^) and the metal is retained in the acceptor solution.

To assemble the EME system, a 4 cm piece of hollow fibre was cut out and one end was closed with a soldering gun. Then, the fibre was immersed for one minute into the organic phase to impregnate the pores. After that, the acceptor solution was introduced into the lumen of the hollow fibre with a Hamilton syringe and the open end of the fibre was connected to a pipette tip and immersed into the sample. Finally, one electrode, the cathode, was immersed into the sample, and the other one, the anode, into the acceptor solution inside the lumen of the hollow fibre.

Once the extraction was finished, the closed end of the hollow fibre was cut to recover the internal solution, which was flushed into a vial using a syringe and weighted to control the stability of the membrane. Three replicates were performed for each EME experiment and then analysis of silver in the internal solution was performed by graphite furnace atomic absorption spectrometry using the temperature program shown in Table 1.

### 2.4. Optimization

Statgraphics Centurion XVII software was used to perform the experimental design. First, a fractional factorial design was carried out to establish the most influential factors. The selected variables for the fractional factorial design were Cyanex 471x concentration in the organic phase, sodium thiosulphate concentration in the acceptor solution, nitrate concentration in the sample, the electric potential, the extraction time and the stirring rate. The pH of the acceptor solution was not included, since previous studies demonstrated that the extraction system was independent of the pH of the samples [32]. The levels of the factors are shown in Table 2. In order to reduce the number of required experiments, a half-fractional factorial design (2^6−1^), with two central points and two blocks, was executed. A total of sixty-eight experiments were run randomly to minimize the effect of uncontrolled factors. Once the most influential variables were determined, a Draper–Lin response surface design was applied to obtain the optimal values for each of these variables.

Enrichment factor (EF) was used as response variable in the optimization process and it was calculated using Equation (1):

(1) $$\mathrm{EF}=\frac{{\left[\mathrm{Ag}\right]}_{a}}{{\left[\mathrm{Ag}\right]}_{s}}$$

where [Ag]_a_ is the silver concentration in the acceptor solution after extraction and [Ag]_s_ is the silver concentration in the sample solution before the extraction.

### 2.5. Effect of Saline Matrix

**Figure 1:**
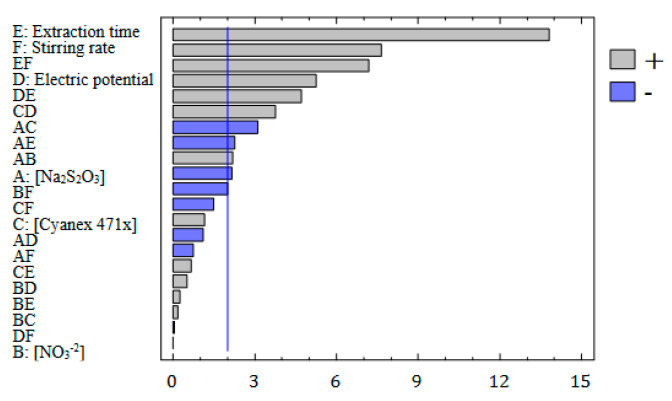
Standardized (*p* = 0.05) Pareto chart, representing the estimated effects of main factors and interactions on silver preconcentration.

To evaluate the applicability of the optimized system to silver analysis in saline samples, synthetic samples with a silver concentration of 0.2 mg L^−1^ and sodium chloride concentrations in the range of 5–35 g L^−1^ were extracted using the optimized system. Afterwards, an EF was calculated using two real seawater samples in order to take into account the complete effects of saline matrix components before the analysis of real samples. Previous studies have shown that the enrichment factor for real samples can be lower than that obtained for synthetic samples due to the simultaneous transport of other cations present in the samples when non-selective extractants (such as DEHPA) are used [33]. These samples were taken in the Bay of Cádiz (see Figure 1) using clean sampling techniques and the silver concentration in the samples was determined by ICP-MS after liquid–liquid extraction with APDC/DDDC [34]. Then, samples were extracted using the optimized EME system and the EF for real seawater samples was calculated using Equation (1). The concentration of silver in the acceptor solution was determined by GFAAS, under the conditions described in Table 1.

### 2.6. Application to Real Samples

Three real seawater samples were taken in the Bay of Cadiz (see Figure 1) to evaluate the applicability of the EME system. The samples were collected in preconditioned low-density polyethylene bottles using clean sampling techniques, filtered using a 0.45 μm pore size nylon filter and acidified [34,35]. After extraction, silver concentration in the received solution was analysed using GFAAS (Table 1) and silver concentration in the samples was calculated using the EF obtained from real seawater samples as described before.

The results obtained with the proposed method were statistically compared with those obtained by a well-established methodology based on liquid–liquid extraction with APDC/DDDC and ICP-MS determination [34].

## 3. Results and Discussion

### 3.1. Optimization

#### 3.1.1. Screening of Factors

**Figure 2:**
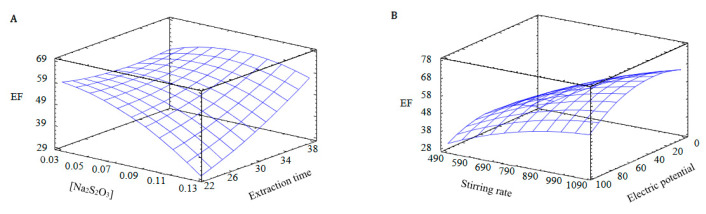
Response surface for Draper–Lin design: (**A**): electric potential, 50 V; stirring rate, 750 rpm. (**B**): extraction time, 30 min; [Na_2_S_2_O_3_] 0.075 N.

The conditions for each experiment produced by the fractional factorial design (described in the experimental section) are shown in Table S1 (Supplementary Materials), as well as the response obtained for each one. To select the most influential variables on the enrichment factor, an analysis of the variance (ANOVA) was performed. The results are summarized in a Pareto chart (Figure 2), where the vertical line defines which variables can be considered statistically significant for a probability level of 95%. As can see in Figure 2, the non-chemical factors presented high effects in the EME system, the extraction time and the stirring rate being the most influential variables, as well as their interaction, followed by the electric potential. These things considered, these variables showed a positive correlation with the enrichment factor. As expected, increasing the extraction time implies more contact time between the sample and the organic phase, and silver transport is favoured. In the case of stirring rate, the increase of enrichment factor is related to the improvement of diffusion processes due to the reduction of the diffusion layer [6]. Finally, other studies have pointed out that the transport of the analytes is favoured by increasing the electric potential as long as electrolysis is avoided [4,6,21,25]. In this sense, it must be noted that the interaction between extraction time and electric potential also has an important effect due to a combination of both factors determining the occurrence of electrolysis and Joule’s effect.

In the case of chemical factors, only the concentration of sodium thiosulphate can be considered as a significant variable, but in less extension than non-chemical factors. In any case, in the studied range, lower concentrations of sodium thiosulphate seem to improve the enrichment factor, as can be concluded from the negative correlation between these variables. This behaviour has been also observed in previous studies with other HF-LPME systems without the application of an electric potential, suggesting that low concentrations of sodium thiosulphate are enough for transport occurrence [36].

Finally, despite the Cyanex 471x concentration seeming to have a minor influence on the extraction, several studies have shown that the use of carriers improves the transport of analytes in EME [37]. In this case, the interaction of this factor with the electric potential showed a significant, but minor and negative, influence on the enrichment factor. In this way, Goodarzi et al. observed that the presence of Aliquat-336 as a carrier improve the Cr extraction by EME, but the extraction decreased as Aliquat-336 concentration increased higher than 2.5% *w*/*v* [38]. They attributed this behaviour to the increase in the density of the organic phase, and the decrease of the electric resistance of SLM, allowing an increase in the electric current through the extraction system and reducing the stability of the EME. Then, although the presence of a carrier is necessary to improve the transport, high concentrations in combination with the application of electric potential could lead to lower extraction factors.

#### 3.1.2. Response Surface Methodology (RSM)

Once the most influential variables were selected, a Draper–Lin design was used to obtain the optimum values of these variables. In this case, the studied ranges were selected taking into account the positive or negative correlations of these variables with the enrichment factor obtained from the fractional factorial design (Table 3). The experimental conditions obtained from the Draper–Lin design as well as the enrichment factor achieved for each experiment can be observed in Table S2 (Supplementary Materials).

In Figure 2, the analytical response (EF) is represented as a function of two experimental factors, maintaining constant the values of the other factors, in order to understand the behaviour of each one. Figure 2A shows the EF as a function of the extraction time and the thiosulphate concentration in the acceptor solution, maintaining the stirring speed and the electric potential at 750 rpm and 50 V, respectively. As can be observed, the maximum EF is associated with low thiosulphate concentrations and long extraction times, in agreement with the results from the fractional factorial design. Additionally, Figure 2B shows the EF as a function of the stirring rate and the electric potential, maintaining the extraction time and the thiosulphate concentration at 30 min and 0.075 N, respectively. In this case, the best EF values are related to high values of stirring rates and medium values of electric potential. As was mentioned before, the electric potential has a positive effect on the enrichment factor, but too high values of electric potential can produce a heating of the system, and a reduction in the enrichment factor due to electrolysis could appear [6].

From the results obtained, the optimum values for each variable were a thiosulphate concentration of 0.03 N, an electric potential of 74 V, a stirring rate of 1000 rpm and an extraction time of 22 min. Under these conditions, the experimental EF was 49.91 ± 3.95. If comparing this system for Ag extraction with a previous HF-LPME system based on the same transport mechanism but in the absence of electric potential, the extraction time needed to obtain a similar EF was 120 min. Thus, the rate between the EF and the optimum extraction time, as a measurement of the efficiency of the system, was more than four times better when EME was used (133.5 h^−1^ vs. 30 h^−1^) [29].

### 3.2. Effect of Saline Matrix

**Figure 3:**
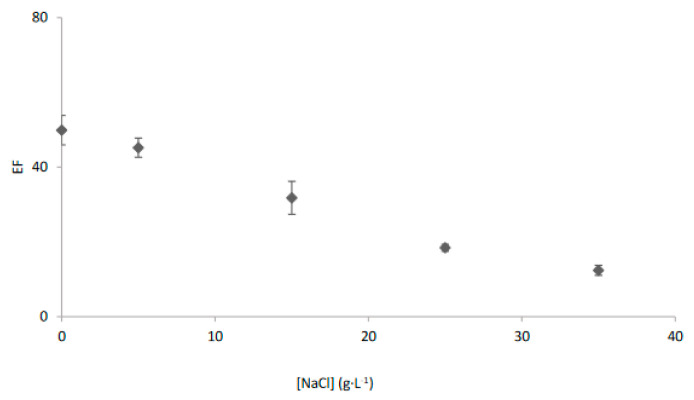
Salinity effect on the transport of Ag^+^ with the EME system and Cyanex 471x in kerosene as organic phase.

To determine the applicability of the system to saline water samples, the effect of salinity in the enrichment factor was evaluated. As can be seen in Figure 3, the enrichment factor decreased as salinity increased achieving a value of 12.4 ± 1.3 for a typical salinity of seawater. This behaviour has been previously related to the formation of silver chloride complexes that could be not extracted by Cyanex 471x due to its polarity [27,28,33,39].

**Figure 4:**
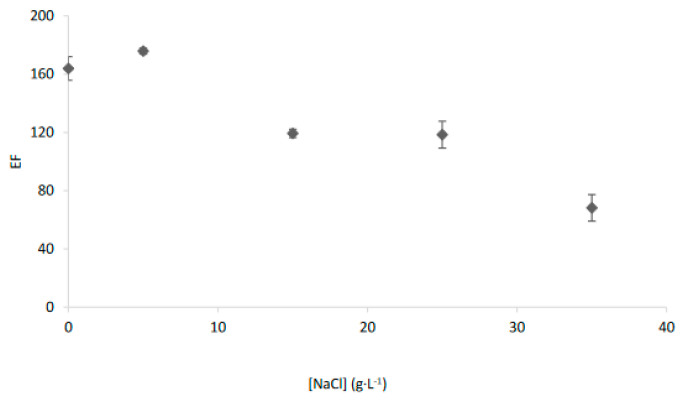
Salinity effect on the transport of Ag^+^ with the EME system and Cyanex 471× in 1-octanol as organic phase.

Then, in order to facilitate the transport of these chemical species which were not transported through a kerosene-based membrane, additional experiments were carried out by increasing the polarity of the organic membrane according to previous studies [5]. Thus, by using 1-octanol as organic solvent and increasing extraction time to 30 min, an average EF of 68.2 ± 9.1 was obtained for saline samples with 35 g L^−1^ of NaCl, as may be seen in Figure 4.

### 3.3. Applicability of EME System

Finally, the system was applied to the determination of silver in several real seawater samples, at the ultra-trace level. As explained in the experimental section, an EF to real seawater sample was calculated from two samples of seawater from the Bay of Cádiz using both the reference method and the optimized EME system, obtaining a value of 33.7 ± 13.8. Then, this factor was applied to calculate the concentration of silver in three other seawater samples. The results obtained after the EME process were compared with the reference method as shown in Table 4. As observed, no significant differences between the silver concentrations obtained by the proposed EME and those obtained by liquid–liquid extraction with ICP-MS were observed based on a Student’s *t* test (*n* = 3, α = 0.05).

## 4. Conclusions

The application of a multivariate approach allowed determining the most influential factors in EME systems. From the results of the fractional factorial design, non-chemical factors such as electric potential, extraction time and stirring rate appear to be the most significant, even though the presence of sodium thiosulphate in the acceptor solution and Cyanex 471x in the organic phase is necessary for the transport mechanism occurrence. Then, a Draper–Lin design was performed with the most influential factors and a maximum experimental enrichment factor of 49.91 ± 3.95 was achieved after 22 min under the optimum conditions.

Nevertheless, the application of the EME system to saline waters required a more polar organic phase in order to reduce the decrease of the enrichment factor observed when the salinity of the sample increases. Then, using 1-octanol as a solvent in the organic phase an enrichment factor of 33.7 ± 13.8 was achieved for real seawater samples after 30 min.

In both cases, the extraction times were very short and always shorter than those required in similar systems in the absence of electric potential. Using this enrichment factor the system was applied to analyse silver in three real seawater samples and no significant differences were found in comparison with the results obtained with a well-established methodology, proving the applicability of the systems to analysing ultra-trace silver concentrations in real seawater samples.

## Figures and Tables

**Table 1:** Temperature program for the analyses of Ag^+^ in the acceptor solution by graphite furnace atomic absorption spectroscopy.

Step	Temperature (°C)	Ramp (°C/s)	Hold (s)
Dry	80	6	20
Dry	90	3	20
Dry	110	5	10
Pyrolysis	350	50	20
Pyrolysis	1000	300	10
Atomization	1900	1500	3
Clean-out	2450	500	4

**Table 2:** Levels of the factors for the fractional factorial design.

Factors	Levels
[Na_2_S_2_O_3_]	0.05–1 N
[KNO_3_]	0.05–1 M
[Cyanex 471x]	0.05–1 M
Electric potential	0–30 V
Extraction time	5–20 min
Stirring rate	300–1000 rpm

**Table 3:** Levels of the factors for the Draper–Linn design.

Factors	Levels
[Na_2_S_2_O_3_]	0.05–0.1 N
Electric potential	25–75 V
Extraction time	25–35 min
Stirring rate	600–900 rpm

**Table 4:** Application of EME system to the analysis of silver in seawater samples.

Sample	^1^ [Ag]_ref_. ng·L^−1^	[Ag]_EME_ ng·L^−1^	%Recovery	%Error	t	*t*-Test
1	6.99 ± 2.71 ^1^	6.02 ± 0.81	86.08	−13.87	0.60	Accepted ^a^
2	17.58 ± 1.40 ^1^	18.83 ± 1.89	107.15	−7.11	0.87	Accepted ^b^
3	28.61 ± 6.52 ^1^	22.68 ± 6.23	79.27	20.73	1.14	Accepted ^a^

^a^ t_4_ = 2.78 (α = 0.05); ^b^ t_3_ = 3.18 (α = 0.05); ^1^ ICP-MS.

## Supplementary Materials

The following supporting information can be downloaded at: https://www.mdpi.com/article/10.3390/membranes13010011/s1, Table S1: Fractional factorial design for the optimization of an EME system for silver preconcentration, Table S2: Draper-Lin design for the optimization of an EME system for silver preconcentration.
