# Supplementary material for: Analytical Performance of Electromembranes as a Tool for Nanoconcentrations of Silver in Waters
Source: Membranes (Basel). 2022 Dec 21;13(1):11. doi: 10.3390/membranes13010011 (PMC9867316; doi:10.3390/membranes13010011)
Supplement: Supplementary file 1 [file membranes-13-00011-s001.zip › membranes-2089091-supplementary.pdf]

Table S1. Factorial fractional design for the optimization of an EME system for silver preconcentration. (N: number of experiment; EF: enrichment factor).

Block	N	$[Na_2S_2O_3]$ (N)	$[KNO_3]$ (M)	[Cyanex 471x] (M)	Electric potential (V)	Extraction time (min)	Stirring rate (rpm)	EF
1	1	0.05	0.05	0.05	0	5	300	3.9
1	2	1	1	1	30	5	300	2.4
1	3	1	1	1	30	20	1000	14.5
1	4	1	0.05	0.05	30	5	300	4.8
1	5	0.05	1	0.05	0	5	1000	2.4
1	6	1	1	0.05	0	5	300	2.9
1	7	1	0.05	1	0	5	300	3.0
1	8	0.05	1	1	30	20	300	35.4
1	9	0.525	0.525	0.525	15	12.5	650	12.2
1	10	0.05	0.05	0.05	30	5	1000	3.2
1	11	1	1	0.05	0	20	1000	33.2
1	12	0.05	0.05	1	30	5	300	7.2
1	13	0.05	1	1	0	20	1000	19.3
1	14	0.05	1	0.05	0	20	300	19.4
1	15	0.05	0.05	1	0	5	1000	4.1
1	16	0.05	0.05	0.05	0	20	1000	34.6
1	17	1	1	0.05	30	20	300	24.1
1	18	0.05	1	0.05	30	5	300	4.0
1	19	0.525	0.525	0.525	15	12.5	650	16.7
1	20	0.05	1	1	30	5	1000	7.3
1	21	1	1	1	0	5	1000	3.8
1	22	0.05	1	1	0	5	300	3.8
1	23	1	1	1	0	20	300	11.4
1	24	1	0.05	1	30	20	300	7.5
1	25	0.05	1	0.05	30	20	1000	19.4
1	26	1	0.05	0.05	0	20	300	7.7
1	27	1	0.05	1	30	5	1000	15.5
1	28	0.05	0.05	0.05	30	20	300	13.9
1	29	1	0.05	0.05	30	20	1000	34.6
1	30	1	0.05	1	0	20	1000	17.4
1	31	1	1	0.05	30	5	1000	6.7
1	32	0.05	0.05	1	0	20	300	8.4
1	33	0.05	0.05	1	30	20	1000	5.5
1	34	1	0.05	0.05	0	5	1000	0
2	35	0.05	0.05	0.05	0	5	300	0
2	36	1	1	1	30	5	300	0
2	37	1	1	1	30	20	1000	6.7
2	38	1	0.05	0.05	30	5	300	0
2	39	0.05	1	0.05	0	5	1000	0
2	40	1	1	0.05	0	5	300	0
2	41	1	0.05	1	0	5	300	0
2	42	0.05	1	1	30	20	300	0
2	43	0.525	0.525	0.525	15	12.5	650	1.2
2	44	0.05	0.05	0.05	30	5	1000	3.3
2	45	1	1	0.05	0	20	1000	3.2
2	46	0.05	0.05	1	30	5	300	0.5
2	47	0.05	1	1	0	20	1000	2.4
2	48	0.05	1	0.05	0	20	300	3.1
2	49	0.05	0.05	1	0	5	1000	0

2	50	0.05	0.05	0.05	0	20	1000	4.4
2	51	1	1	0.05	30	20	300	1.7
2	52	0.05	1	0.05	30	5	300	0
2	53	0.525	0.525	0.525	15	12.5	650	0.8
2	54	0.05	1	1	30	5	1000	0.8
2	55	1	1	1	0	5	1000	0
2	56	0.05	1	1	0	5	300	0
2	57	1	1	1	0	20	300	0.8
2	58	1	0.05	1	30	20	300	4.7
2	59	0.05	1	0.05	30	20	1000	3.0
2	60	1	0.05	0.05	0	20	300	2.8
2	61	1	0.05	1	30	5	1000	3.1
2	62	0.05	0.05	0.05	30	20	300	2.8
2	63	1	0.05	0.05	30	20	1000	3.5
2	64	1	0.05	1	0	20	1000	1.3
2	65	1	1	0.05	30	5	1000	0
2	66	0.05	0.05	1	0	20	300	1.3
2	67	0.05	0.05	1	30	20	1000	5.6
2	68	1	0.05	0.05	0	5	1000	0

Table S2. Draper-Lin design for the optimization of an EME system for silver preconcentration. (N: number of experiment; EF: enrichment factor).

Block	N	[$Na_2S_2O_3$] (N)	Extraction time (min)	Electric potential (V)	Stirring rate (rpm)	EF
1	1	0.05	25	75	600	33.0
1	2	0.08	30	50	498	34.4
1	3	0.08	30	50	750	63.0
1	4	0.1	35	25	600	46.2
1	5	0.05	25	25	600	36.4
1	6	0.08	30	8	750	61.8
1	7	0.08	30	50	1002	59.6
1	8	0.08	30	50	750	45.1
1	9	0.08	38	50	750	62.7
1	10	0.03	30	50	750	54.0
1	11	0.08	22	50	750	45.4
1	12	0.1	25	25	900	45.4
1	13	0.12	30	50	750	50.8
1	14	0.1	35	75	600	63.3
1	15	0.05	35	25	900	52.8
1	16	0.1	25	75	900	51.9
1	17	0.05	35	75	900	60.8
1	18	0..8	30	92	750	37.6
2	19	0.05	25	75	600	41.4
2	20	0.08	30	50	498	47.8
2	21	0.08	30	50	750	60.6
2	22	0.1	35	25	600	46.9
2	23	0.05	25	25	600	34.2
2	24	0.08	30	8	750	56.4
2	25	0.08	30	50	1002	68.9
2	26	0.08	30	50	750	39.0
2	27	0.08	38	50	750	65.7
2	28	0.03	30	50	750	60.5
2	29	0.08	22	50	750	58.2
2	30	0.1	25	25	900	49.2
2	31	0.12	30	50	750	40.4
2	32	0.1	35	75	600	53.7
2	33	0.05	35	25	900	66.6
2	34	0.1	25	75	900	49.7
2	35	0.05	35	75	900	62.6
2	36	0.08	30	92	750	41.6
